# Validity of Wearable Inertial Sensors for Gait Analysis: A Systematic Review

**DOI:** 10.3390/diagnostics15010036

**Published:** 2024-12-27

**Authors:** Giuseppe Prisco, Maria Agnese Pirozzi, Antonella Santone, Fabrizio Esposito, Mario Cesarelli, Francesco Amato, Leandro Donisi

**Affiliations:** 1 Department of Medicine and Health Sciences, University of Molise, 86100 Campobasso, Italy; g.prisco2@studenti.unimol.it (G.P.); antonella.santone@unimol.it (A.S.); 2 Department of Advanced Medical and Surgical Sciences, University of Campania Luigi Vanvitelli, 80138 Naples, Italy; mariaagnese.pirozzi@unicampania.it (M.A.P.); fabrizio.esposito@unicampania.it (F.E.); 3 Department of Engineering, University of Sannio, 82100 Benevento, Italy; mcesarelli@unisannio.it; 4 Department of Information Technology and Electrical Engineering, University of Naples Federico II, 80125 Naples, Italy; framato@unina.it

**Keywords:** agreement, biomechanics, gait analysis, inertial measurement units, wearable sensors

## Abstract

**Background/Objectives**: Gait analysis, traditionally performed with lab-based optical motion capture systems, offers high accuracy but is costly and impractical for real-world use. Wearable technologies, especially inertial measurement units (IMUs), enable portable and accessible assessments outside the lab, though challenges with sensor placement, signal selection, and algorithm design can affect accuracy. This systematic review aims to bridge the benchmarking gap between IMU-based and traditional systems, validating the use of wearable inertial systems for gait analysis. **Methods**: This review examined English studies between 2012 and 2023, retrieved from the Scopus database, comparing wearable sensors to optical motion capture systems, focusing on IMU body placement, gait parameters, and validation metrics. Exclusion criteria for the search included conference papers, reviews, unavailable papers, studies without wearable inertial sensors for gait analysis, and those not involving agreement studies or optical motion capture systems. **Results**: From an initial pool of 479 articles, 32 were selected for full-text screening. Among them, the lower body resulted in the most common site for single IMU placement (in 22 studies), while the most frequently used multi-sensor configuration involved IMU positioning on the lower back, shanks, feet, and thighs (10 studies). Regarding gait parameters, 11 studies out of the 32 included studies focused on spatial-temporal parameters, 12 on joint kinematics, 2 on gait events, and the remainder on a combination of parameters. In terms of validation metrics, 24 studies employed correlation coefficients as the primary measure, while 7 studies used a combination of error metrics, correlation coefficients, and Bland–Altman analysis. Validation metrics revealed that IMUs exhibited good to moderate agreement with optical motion capture systems for kinematic measures. In contrast, spatiotemporal parameters demonstrated greater variability, with agreement ranging from moderate to poor. **Conclusions**: This review highlighted the transformative potential of wearable IMUs in advancing gait analysis beyond the constraints of traditional laboratory-based systems.

## 1. Introduction

Human locomotion is a sophisticated process requiring complex interplay between multiple systems, including skeletal alignment, joint mobility, neuromuscular regulation, and the biomechanical forces that direct movement [1,2]. Pathological conditions such as congenital deformities, developmental issues, traumatic injuries, or degenerative disorders can affect these systems, leading to decreased walking efficiency and reduced mobility [3,4,5,6]. Therefore, the possible comprehensive gait assessment using gait analysis (GA), which systematically examines human movement, is crucial in several fields, including sports, clinical diagnoses, physical ergonomics, and rehabilitation [7,8,9,10,11]. In sports, GA helps in determining better athletic performance, preventing injuries, as well as developing tailored training programs [12,13]. From a clinical point of view, GA allows identifying abnormalities, monitoring recovery, and evaluating fall risk, especially among the elderly [14,15]. At the same time, for rehabilitation purposes, it provides therapists with critical data to track patient progress, enabling them to modify treatment plans based on mobility improvements [16,17].

**Figure 1:**
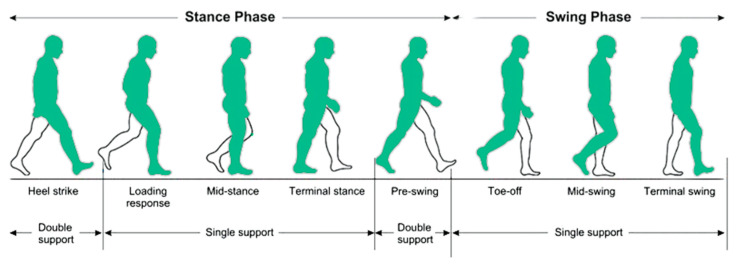
Gait phases in a single gait cycle.

Human walking consists of a cyclical motion divided into distinct gait phases: the stance phase (60% of the cycle), where the foot remains on the ground, and the swing phase (40% of the cycle), where the foot moves forward [18]. These phases, including heel strike, toe-off, and various stages of swing, form a coordinated cycle of leg and foot movements (Figure 1). Thus, to provide deeper insights into human movement, quantitative GA focuses on measuring, describing, and evaluating several parameters, including kinematic, kinetic, and spatiotemporal (ST) metrics [19].

The origins of GA date back to the 19th century, with pioneers such as Étienne-Jules Marey and Eadweard Muybridge using sequential photography to study motion [20]. The 20th century marked a significant milestone in the integration of biomechanics with GA, highlighted by the introduction of force plates and multi-camera optical motion capture (OMC) systems [21,22,23,24]. These innovations enabled accurate measurements of forces and three-dimensional movement, establishing OMC systems as a practical gold standard in the GA field [25,26]. OMC systems offer high accuracy in motion capture, making them ideal for advanced biomechanical studies in various fields, from sports medicine and rehabilitation to scientific research [27,28]. In addition, OMC systems excel in controlled (with sufficient space and good lighting) and unobstructed environments, such as motion analysis labs, where precise tracking is essential [29]. However, conventional OMC systems are often more expensive and complex to install, resulting in lengthy set-up times, as well as space issues, requiring specialized structures, which may also hinder their ability to accurately reflect real-world walking scenarios [30].

In recent years, the emergence of wearable technologies, including inertial measurement units (IMUs) which combine accelerometers, gyroscopes, and magnetometers, has revolutionized GA. These devices facilitate assessments outside traditional lab settings, providing more realistic evaluations of human movement and being very useful in the context of telemedicine for remote patient monitoring. Indeed, inertial systems are typically more portable and affordable than optical systems, enabling motion analysis in any environment—including outdoors or in confined spaces—without requiring cameras or specialized lighting. Therefore, advances in miniaturization and accessibility have fostered a shift in movement biomechanics, making wearable sensors a viable alternative to established OMC systems [31,32,33]. The integration of these wearable inertial systems with advanced signal processing algorithms for gait events (GEs) detection holds significant promise for enhancing the extraction of kinematic parameters and transforming how movement is monitored [34,35,36,37,38,39]. The primary limitation of inertial systems is sensor drift, which causes error accumulation and progressively reduces tracking accuracy over time. Moreover, IMUs are highly sensitive to sensor placement, considering factors such as soft-tissue artefacts, muscle movement, and attachment to non-bony areas introducing significant noise and compromising data reliability. Furthermore, IMUs cannot directly measure kinetic variables (e.g., joint moments or power) and may be influenced by external magnetic or metallic interference, which can occasionally result in system failure. In contrast, OMC systems offer highly accurate and direct measurements of both kinematic and kinetic variables by tracking reflective markers in controlled environments. However, their substantial infrastructure requirements limit their feasibility in real-world settings. Additionally, IMUs are less accurate in capturing complex movements, as they may struggle to detect subtle changes or rotations as accurately as OMC systems.

Despite the growing adoption of wearable inertial sensors and specialized motion analysis algorithms, there is still a lack of systematic literature review studies validating their effectiveness. Possible inconsistencies in the adoption of different wearable inertial sensors may indeed lead to potential variability in gait parameter estimates, which may arise from differences in sensor placement and in the inertial signals used to calculate kinematic parameters [40,41,42].

Therefore, in the wake of the increasing use of wearable inertial sensors in the field of GA, this systematic review aims to address, possibly filling, gaps of inconsistency in the literature, proposing a systematic analysis of the validity of wearable sensors, both prototype and commercial ones, comparing the benchmarking with OMC systems, which can be considered the gold standard also for clinical applications.

## 2. Research Strategy

A systematic review involves the rigorous selection, evaluation, and synthesis of studies on a defined subject [43]. This review adheres to the guidelines set by the Preferred Reporting Items for Systematic Reviews and Meta-Analyses (PRISMA) [44] (the checklist is included in the Supplementary Materials).

### Search Methodology and Study Selection

The literature search was conducted by searching for documents in the Scopus database and was limited to English documents published between 2012 and 2023. The database was queried using the following keyword structure: ((“agreement”) or (“benchmarking”) or (“validity”)) and ((“optoelectronic system”) or (“stereophotogrammetry”)) and ((“wearable sensor”) or (“imu”) or (“inertial measurement unit”) or (“accelerometer”)) and ((“gait analysis”) or (“gait”)).

In order to simplify our research, the exclusion criteria were:

- manuscripts not published in English;
- conference reviews, reviews, and book chapters;
- papers not available.

For the screening process involving titles, abstracts, and full texts, the following exclusion criteria were established:

- papers assessing gait parameters without utilizing wearable inertial sensors;
- papers comparing wearable inertial sensors with OMC systems for tasks not related to gait analysis;
- papers that do not include agreement studies;
- papers using wearable inertial sensors for gait assessment that do not compare results with OMC systems.

Documents were screened by first assessing the content of the titles and abstracts. If any documents did meet the inclusion criteria at this stage, a full-text evaluation was conducted. Following the completion of the initial search of electronic database, one reviewer (G.P.) examined the titles and abstracts of the identified articles to determine their eligibility for inclusion in the review. Full-text evaluations of potentially relevant articles were conducted independently by two reviewers (G.P. and M.A.P.).

**Figure 2:**
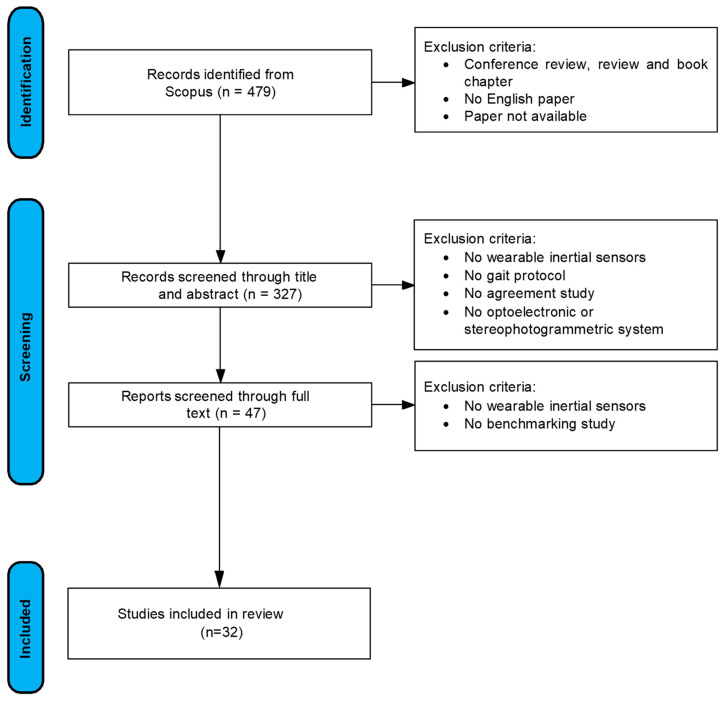
Summary review workflow.

The PRISMA workflow is illustrated in Figure 2, which also indicates the number of documents included in this systematic review.

## 3. Main Findings and Argumentation

**Figure 3:**
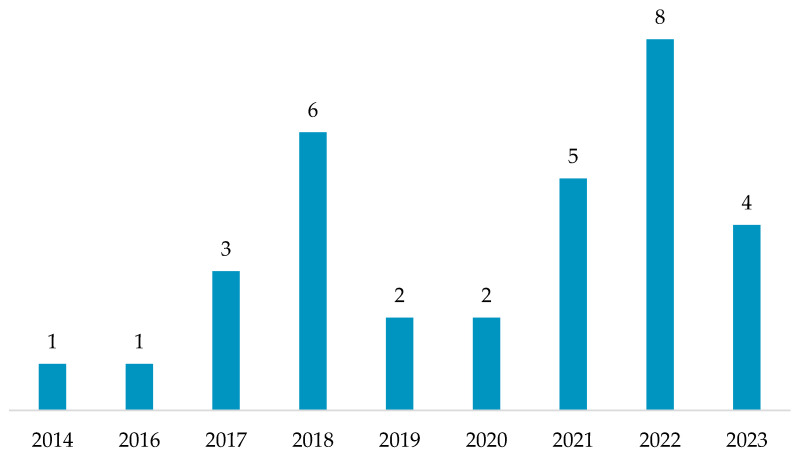
Distribution of papers over time.

This systematic review encompasses 32 studies, published between 2014 and 2023. Publication activity reached its highest point in 2022, as depicted in Figure 3. This surge highlights the rising interest in applying IMUs for GA from both practical and scientific research perspectives. This demonstrates the growing role of IMUs in clinical practice, particularly in assessing gait parameters. Their portability and relatively low cost make them ideal for tracking rehabilitation progress or identifying motor impairments without relying on complex motion analysis labs.

Data collection, organization of tabular information, and the creation of graphics and charts were conducted using Microsoft Excel 2021. The papers were examined across several key aspects: the aim of the study, the participants involved, and the tasks performed; the type of wearable inertial system used, including the number of the devices used and their placement on the body; the OMC system, including the number of cameras; the extracted kinematic and kinetic parameters; the statistical methodologies employed; and the results obtained for each study. Table 1 presents the studies, listed in chronological order by publication year.

## 4. Wearable Inertial Systems and Study Population

The development of wearable devices has rapidly advanced due to the introduction of novel sensors and technologies [77]. These innovations enable continuous monitoring of various physiological parameters, demonstrating versatility across a wide range of healthcare applications, including the management of chronic and degenerative diseases, as well as other medical conditions [78,79,80,81,82,83,84]. The reviewed studies focused on wearable inertial systems and their placement on the human body to detect ST and kinematic gait parameters to establish a benchmark with gold-standard OMC systems.

**Figure 4:**
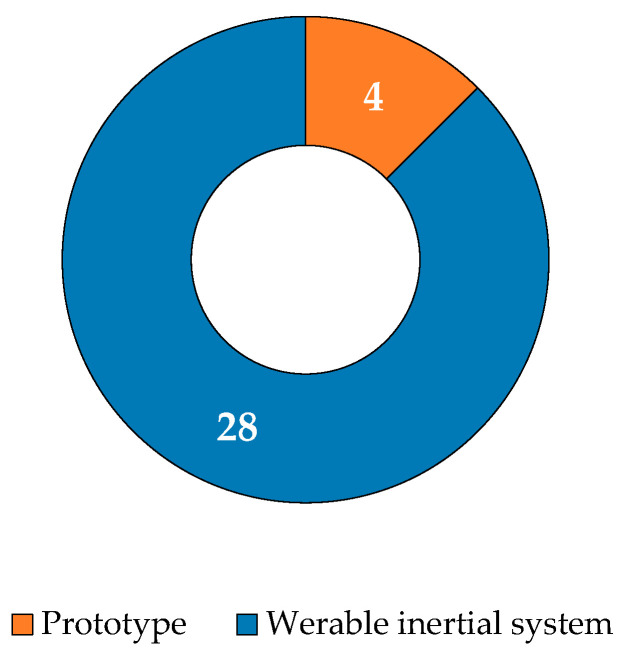
Distribution of wearable sensors: prototypes and commercial wearable inertial systems.

Two categories of devices were considered: (1) prototypes, representing experimental configurations not commercially available, and (2) commercial devices, referring to those already widely available (Figure 4). Among the reviewed studies, 4 out of 32 papers utilized a prototype device. Pepa et al. [47] investigated the accuracy of different spatial gait parameter estimation methods using the iPhone 4s accelerometer, positioned on the lumbar region, and compared the results with OMC data. Koska et al. [50] assessed the accuracy of kinematic data related to human running obtained from brief sequences of shoe-mounted IMU sensors, comparing the results with those from an OMC system. Amitrano et al. [58] evaluated a prototype system that combined e-textile sensor socks with ankle-mounted IMUs to assess both postural and ST gait parameters. Hellec et al. [71] aimed to determine the concurrent validity of ST parameters captured by an IMU integrated into smart glasses, compared with an OMC system.

Other studies utilized commercial inertial systems, enrolling both healthy subjects and patients suffering from specific impairments. Of these studies, 10 out of 32 works tested the wearable inertial systems during gait tasks on participants with specific impairments. The distribution of subject impairments considered across the studies is presented in Table 2. Cimolin et al. [48] validated ST parameter estimates during level walking using a single IMU positioned on the lower trunk of both obese and normal-weight adolescents. Pham et al. [49] assessed an algorithm for step detection in 11 participants with Parkinson’s disease (PD) during turning and non-turning episodes, utilizing an IMU placed on the lumbar region. Kleiner et al. [51] compared total times from the timed up and go (TUG) test in 30 PD participants, measuring outcomes with a wearable tri-axial IMU against an OMC system. Zago et al. [53] evaluated the validity of two systems for measuring ST gait parameters (BTS^®^ G-Sensor vs. BTS SMART System) in 22 PD participants. Berner et al. [59] investigated the validity of an IMU system in measuring lower limb kinematics and ST gait parameters with eight HIV-positive and eight HIV-negative participants. Simonetti et al. [63] validated a wearable framework for estimating the center of mass (CoM) acceleration and velocity using one participant with transfemoral amputation. Romijnders et al. [64] assessed GE detection using a shank-mounted IMU in 14 PD participants and 9 stroke (STR) participants. In a subsequent study, the same authors [69] examined the effectiveness of a deep learning approach for detecting GEs from an IMU on the lower leg in 93 participants with various conditions. Ricciardi et al. [73] evaluated the agreement between two systems for measuring ST gait parameters (Opal System vs. BTS SMART System) in 15 participants with progressive supranuclear palsy (PSP). Finally, Brasiliano et al. [75] validated three IMU-based algorithms (shank and foot set-ups) in identifying GEs among children with idiopathic toe walking (ITW).

## 5. Sensor Placement

**Figure 5:**
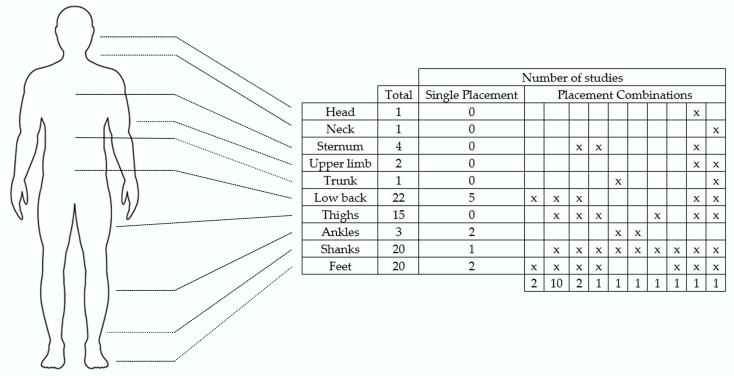
The number of studies that positioned IMUs on specific anatomical locations. The “Single Placement” column represents studies where sensors were located at only one anatomical site. The “Placement Combinations” columns represent studies where sensors were positioned at multiple anatomical locations. Each relevant location is marked with an “x”, and the number of studies utilizing that specific combination is noted at the bottom of each column. The “Total” reflects the cumulative number of studies that placed sensors at the respective anatomical location.

The placement of IMUs offers multiple options, and researchers explored different configurations in gait-related studies, applying sensors to various body segments or combinations of segments. This distribution is highlighted in Figure 5 (one study was excluded from the figure as the sensor was not placed directly on the body but was instead embedded in a pair of eyeglasses [71]). Among the studies analyzed, the lower back was the most frequently used site for lower-body IMU placement in GA (22 studies), followed closely by the feet and shanks (20 studies each). Additionally, 10 out of 32 studies used a single sensor, while the remaining studies employed multiple sensors. The most common multi-sensor set-up involved placing IMUs on the low back, shanks, feet, and thighs (used by ten studies). While these statistics provided insight into current research preferences, it should be noted that they did not necessarily indicate whether these segments delivered the most informative data or simplified the identification of key GEs. Some researchers sought to clarify these considerations further. For instance, Micó-Amigo et al. [46] compared two IMU configurations (placed on the low back and feet) for estimating step time and validated their results against an OMC system. In both configurations, the anteroposterior (AP) acceleration signal was used to identify GEs and calculate step time. The study concluded that step time can be estimated with acceptable accuracy using either a single sensor on the low back or two sensors positioned on the heels, demonstrating flexibility in sensor placement without compromising accuracy. Additionally, Digo et al. [69] conducted a comparative study evaluating three distinct IMU set-ups (ankle, shank, and trunk-mounted IMUs) against a gold-standard OMC system for assessing ST gait parameters in a healthy elderly population. The algorithms for the shank and ankle IMUs focused on identifying GEs from mediolateral (ML) angular velocity signals, while the trunk IMU algorithm analyzed AP acceleration signals. Despite all IMU configurations demonstrating good accuracy, the authors concluded that the trunk-IMU system outperformed both the ankle and shank set-ups in terms of precision in detecting GEs. Moreover, Brasiliano et al. [75] evaluated the effectiveness of three IMU-based algorithms in detecting GEs in children with ITW. The study compared two setups: one where the IMUs were placed on the shanks and another where they were mounted on the feet. All algorithms were applied to the ML angular velocity signals. The authors concluded that the IMU-foot algorithm was the best for identifying heel strikes and estimating ST parameters, while the IMU-shank algorithm excelled at identifying GEs. Finally, Pepa et al. [47] evaluated three algorithms for detecting GEs using a smartphone placed on the low back to assess the potential of smartphones in estimating gait parameters. All methods relied on AP acceleration signals, with results compared against the gold-standard OMC system. The sensor placement on the low back did not negatively impact the precision of ST parameter estimations. The results indicated a high accuracy in estimating GEs using the smartphone, confirming its suitability for gait monitoring. Unlike all the other items, only one involved the use of an IMU placed on the glasses [71]. The authors developed a methodology based on the vertical acceleration signal acquired by an IMU placed on the subject’s glasses for the detection of ST parameters. Good agreement was observed between the measures extracted by IMU embedded in the glasses and the ones obtained from the OMC system. However, a challenge was the variability in IMU positioning. Each time the IMU was attached, its location or orientation within the same body segment could differ, leading to inconsistent datasets and results across subjects. This intra-segment placement variability could be mitigated by employing a fixed mount or holder, ensuring consistent sensor positioning for more reliable measurements.

Another factor contributing to variability in results was the choice of signal (acceleration or angular velocity). Gyroscopes, which measure angular velocity, are unaffected by positional translation because the angular velocity of a rigid body remains consistent across any point on the body, assuming the sensor’s orientation relative to the body segment remains stable. Additionally, gyroscopes are not influenced by gravity and are less prone to noise. In contrast, accelerometers tend to be noisier, sensitive to both position and orientation, and susceptible to gravitational effects. Consequently, despite numerous studies, disagreements persist regarding the optimal sensor positioning, orientation, and signal type.

## 6. Gait Task

Most of the studies (22 out of 32) involved a gait task where participants walked at either a self-selected pace or at three different speeds over a predetermined distance. Five additional studies required participants to walk on a treadmill. Two studies, conducted by Kleiner et al. [51] and El Fezazi et al. [74], used the TUG test. The TUG test measures the time taken for an individual to rise from a seated position, walk three meters, turn around, walk back, and sit down. This test is commonly used to assess mobility issues, particularly in individuals with PD, stroke, or other conditions affecting balance and gait [85]. Jordan et al. [60] chose the acceleration–deceleration ability (ADA) test, which consists of a 20 m sprint, followed by rapid linear deceleration after crossing the 20 m line, and then backpedaling to the same line. Romijnders et al. [64] asked participants to complete three distinct tasks: walking at a self-selected speed over a 5 m path, a slalom task (covering 5 m with a cone placed every meter, at a preferred speed), and a Stroop-and-walk trial (walking back and forth along the 5 m path while performing a numerical Stroop test on a handheld mobile device, at a self-selected pace, until the Stroop test was completed) [86]. Finally, Bartoszek et al. [67] enrolled a single participant who performed a 12 m walk at their preferred speed for the Nordic walking gait style.

## 7. Gait Parameters

**Figure 6:**
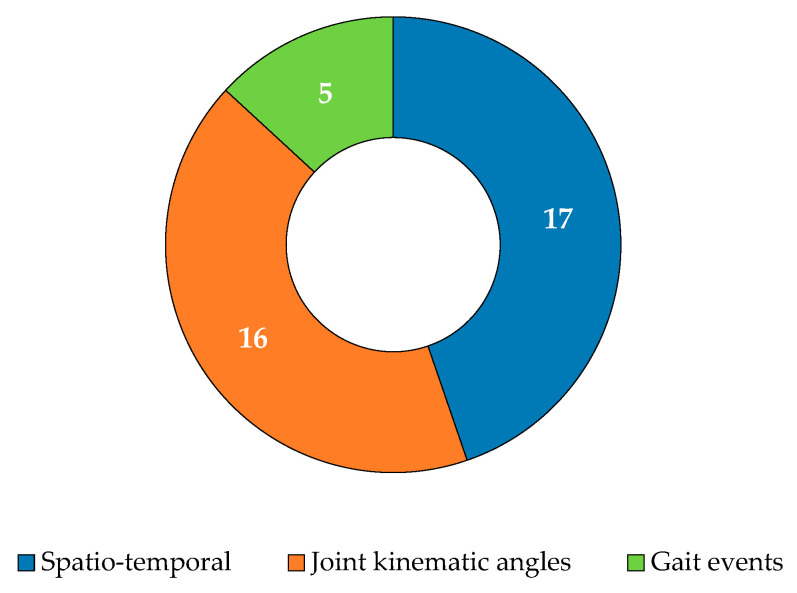
Distribution of gait parameters: spatiotemporal, joint kinematic angles, and gait events.

Gait parameters are essential metrics that characterize human locomotion, offering valuable insights into mobility, efficiency, and injury risk. These parameters are typically classified into three categories: ST, kinematic, and kinetic. ST parameters focus on timing and distances, relying on critical GEs such as heel strike, toe-off, and mid-swing. Kinematic parameters capture the movement of joints and limbs without accounting for forces, while kinetic parameters assess the forces and moments exerted on the body during movement. Notably, no kinetic parameters were reported in the reviewed works. Table 3 presents a detailed breakdown of the parameters measured across the selected studies, while Figure 6 reports the distribution of gait parameters. Joint kinematic angles were recorded 117 times, ST parameters 91 times, center-of-mass parameters 3 times, and gait events 9 times, totaling 233 parameters across 32 studies. Hip flexion was the most frequently measured parameter (ten occurrences), followed by knee flexion (nine occurrences). Hip abduction, hip rotation, ankle flexion, stride length, and speed were each recorded eight times. This is related to the different set-ups (number and positions of sensors that were used).

Of the 32 studies reviewed, 17 focused on calculating ST parameters, while 2 concentrated only on extracting GEs such as heel strike and toe-off, as shown in Figure 6. Pham et al. [47] developed an algorithm that uses an AP acceleration signal from a single IMU placed on the lumbar region to detect GEs. Romijnders et al. [64] designed an algorithm based on angular velocity signals along the ML axis, using two IMUs mounted on the shanks. Both studies were validated by comparing their results with those from two commercial OMC systems, with Pham et al. [47] achieving 90% accuracy and Romijnders et al. [64] achieving 100% accuracy in detecting GEs during non-rotational phases of step detection.

Twelve articles focused exclusively on kinematic parameter estimation, while four studies focused on kinematic parameters coupled with ST parameters. Only one study, Bartoszek et al. [67], investigated the validation of joint kinematic measures for both the lower and upper limbs during the Nordic walking gait. The study found a consistent systematic error across all joint kinematic measurements. Finally, Simonetti et al. [63] focused on two CoM parameters related to gait—CoM velocity and acceleration—to validate a wearable framework in a person with a transfemoral amputation. Strong agreement was obtained by the authors when comparing a network of five IMUs with a gold-standard OMC system.

## 8. Validation Metrics

The present systematic review identified several benchmarking validation metrics, which can be grouped into five categories: correlation coefficients (CC), Bland–Altman (BA) analyses, error metrics (ER), statistical tests (ST), and Passing–Bablok (PB) linear regression.

**Figure 7:**
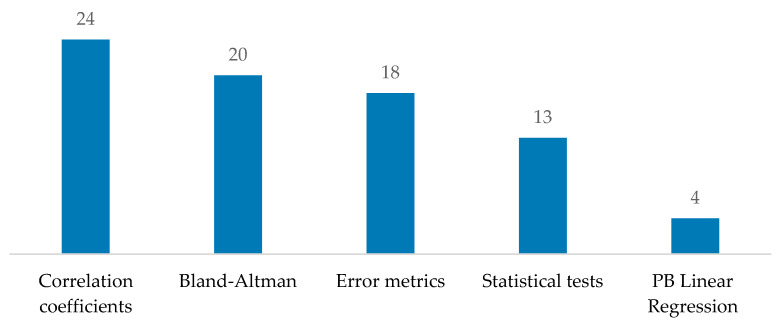
Employed statistical tool distributions.

As reported in Figure 7, in the CC category, the most frequently reported metrics across 24 papers included the Pearson correlation coefficient (PCC), Lin’s concordance correlation (LCC), coefficient of determination (R^2^), Spearman’s rank correlation coefficient (ρ), intraclass correlation coefficient (ICC), and the coefficient of multiple correlation (CMC). The BA approach was the second most widely used, appearing in 20 studies. The ER category was identified in 18 papers and included measures such as mean difference (MD), typical error of estimate (TEE), root mean square deviation (RMSD), root mean square error (RMSE), mean absolute error (MAE), absolute error (ε), absolute percentage error (ε%), absolute difference in range of motion (ΔROM), standardized typical error (SEE), range of motion error (ROME), and non-parametric effect size (ES). The ST category, used in 13 papers, included the paired *t*-Test, Wilcoxon test, ANOVA, and likelihood ratio (LR) test. Finally, the PB linear regression method was reported in four studies.

Overall, CC was the most frequently used metric, followed by BA, ER, ST, and PB linear regression (Figure 7).

While these validation metrics offer insights into current research preferences, individually, they fall short of providing a comprehensive picture of agreement levels. Therefore, combining multiple metrics is essential to achieve more robust and reliable results. Some researchers have explored this approach further to enhance interpretability in validation. For example, Amitrano et al. [58] evaluated the validity of gait parameters measured by the SWEET Sock, a novel wearable device designed for remote health monitoring, using four validation metrics: the paired test (either *t*-Test or Wilcoxon test), PCC, PB linear regression, and BA analysis. The authors found strong agreement for temporal parameters, such as gait cycle time and cadence, but limited agreement for spatial parameters, especially step length. Similarly, El Fezazi et al. [74] developed and validated a method for estimating knee kinematics during the TUG test using IMU devices, comparing it to an optical system through metrics including the paired test, RMSE, PCC, and BA. They concluded that no significant differences were found in the kinematic parameters compared to the reference system, indicating strong agreement between the two methodologies. Ricciardi et al. [73] assessed the agreement between two systems for measuring spatial-temporal gait parameters in patients with PSP by comparing the Opal System and BTS SMART System using the paired test, PB, and BA analyses. They found that while the systems showed general agreement, they were not fully interchangeable due to two types of error: a constant systematic error affecting cadence and gait cycle time, and a proportional error affecting stance phase, swing phase, and stride length. Digo et al. [69] compared three different IMU set-ups—placed on the trunk, shank, and ankle—with an OMC system to evaluate ST parameters in a healthy elderly population. Using metrics such as the PCC, RMSE, and BA analysis, they concluded that all IMU configurations demonstrated good performance in assessing gait. However, the trunk-mounted IMU system showed superior accuracy compared to the shank- and ankle-mounted configurations.

Overall, the combination of validation metrics most frequently used is ER, CC, and BA with seven papers [55,59,62,65,67,68,69].

## 9. Conclusions

This systematic review highlights the transformative potential of wearable IMUs in advancing gait analysis beyond the constraints of traditional laboratory-based systems. This review synthesized findings from studies up to 2023, evaluating the validity of wearable sensors by assessing their benchmarking with gold-standard OMC systems.

The analysis of IMU configurations in the reviewed studies revealed that lower-body placement on the lower back, feet, and shanks is the most common setup, with these sites appearing collectively in over two-thirds of studies. Single-sensor configurations on the lower back proved effective for estimating ST parameters (such as step time and cadence), while multi-sensor setups on the lower back, shanks, feet, and thighs demonstrated superior accuracy for a broader range of gait metrics. Overall, kinematic parameters demonstrated consistently reliable levels of agreement, independently of sensor placement, the number of sensors used, or the validation metrics applied. Among ST parameters, metrics such as cadence, stride time, and stride cycle time demonstrated stronger agreement compared to those describing specific stride cycle phases, such as swing, stance, and double support. Despite high levels of agreement between IMU- and OMC-derived data, several limitations remain in the benchmarking evaluation of the aforementioned systems, especially in confirming the validity of wearable inertial sensors, which would promote their diffusion in clinical practice in the near future.

In conclusion, this review confirmed the promising role of wearable IMUs as viable alternatives to the gold-standard systems (OMCs), particularly in settings where portability and ease of use are paramount. IMUs demonstrated strong agreement with OMC systems in estimating gait parameters. Specifically, kinematic parameters showed good to moderate agreement, while ST parameters exhibited varied results: good agreement for parameters such as cadence and gait cycle time but moderate to poor agreement for parameters associated with gait cycle phases. Therefore, as IMU technology continues to evolve, standardization in positioning, refined signal processing algorithms, and improved validation protocols will be essential to fully realize the potential of IMU-based GA.

Our systematic review identified a key limitation in the heterogeneity of the included studies, particularly in sensor configurations, data processing methods, and validation metrics. This variability hindered direct comparisons and resulted in inconsistent findings. To address these challenges, future research could incorporate meta-analytic approaches to quantitatively synthesize agreement metrics, offering clearer insights into IMU performance trends.

## Figures and Tables

**Table 1:** Analysis of the studies included in this review.

Study	Scope	Population	IMU System (Number and Positioning)	OMC System (Number Cameras)	Gait Parameters	Gait Task	Validation Metrics	Results
Buganè et al. (2014) [45]	Assessing the validity of pelvis kinematics in level walking using a single inertial sensor on the sacrum compared with OMC system	16 volunteer healthy subjects	Free4Act, LetSense Group Srl, Bologna, Italy. (1 IMU: low back)	Vicon Motion Systems, Oxford Metrics Ltd., Oxford, UK. (8 cameras)	Joint kinematic measures of the pelvis	Walking on a 10 m straight pathway at three different speeds	Paired test, R^2^, and PB	The two measurement systems showed good agreement in assessing pelvis kinematics
Micó-Amigo et al. (2016) [46]	Developing and validating a novel algorithm based on two different IMU set-ups (low back and heels) for step duration detection in healthy elderly subjects	20 volunteer healthy subjects	DynaPort^®^ Hybrid, McRoberts B.V., Hague, The Netherlands. (3 IMUs: low back and lateral sides of both heels)	Motion tracking system 3020 Optotrak, Northern Digital Inc., Waterloo, ON, Canada. (3 cameras)	Step time	Walking at self-selected speed along a 5 m pathway	Paired test and ICC	The algorithm can accurately estimate step time in elderly subjects using both IMU configurations when compared to the OMC system
Pepa et al. (2017) [47]	Assessing the smartphone performance in heel strike, step count, step period, and step length estimation compared to ST system using three different kinematic parameters estimation methods	11 volunteer healthy subjects	Prototype (1 IMU: low back)	BTS SMART System, BTS Bioengineering S.p.A., Milan, Italy. (6 cameras)	Step time, step length, step count, heel strike	Walking at three different speeds on a 10 m straight pathway	PCC, ANOVA, and BA	The smartphone demonstrated good accuracy in estimating ST parameters across the three different estimation methods, supporting its suitability for gait monitoring
Cimolin et al. (2017) [48]	Validating the ST parameter estimates in level walking with a single IMU placed on the lower trunk in obese adolescents and normal-weight adolescents	10 obese and 8 normal-weight subjects	BTS^®^ G-Sensor, BTS Bioengineering S.p.A., Milan, Italy (1 IMU: low back)	Vicon Motion Systems, Oxford Metrics Ltd., Oxford, UK. (6 cameras)	Stride length, stride time, stance phase, double support phase, speed, cadence	Walking at self-selected speed on a 10 m walkway	Wilcoxon test, ρ, and BA	No statistical differences were observed between the two systems across all ST parameters analyzed, indicating the effectiveness of the inertial system in evaluating ST parameters
Pham et al. (2017) [49]	Developing and validating an algorithm for step detection during turning and non-turning walking episodes using a single IMU worn at the low back in PD patients and older adults	11 PD participants and 12 older adults	DynaPort^®^ Hybrid, McRoberts B.V., Hague, The Netherlands. (1 IMU: low back)	Vicon Motion Systems, Oxford Metrics Ltd., Oxford, UK. (6 cameras)	Toe-off, heel strike	Treadmill walking for 120 s at self-selected speeds	LR test and BA	Comparable validity was assessed by comparing the IMU-based low back algorithm with an OMC system in PD patients and older adults, which detected 88% of steps during turning episodes
Koska et al. (2018) [50]	Investigating the validity of kinematic measures of human running from shoe-mounted IMU system and compared to OMC system	51 volunteer healthy subjects	Prototype (1 IMU: heel cup of the right shoe)	Motion Capture system MA, Qualisys AB, Göteborg, Danmark. (14 cameras)	ROM of foot	Treadmill running at three different speeds	BA	The disagreement between IMUs and the OMC system suggests that shoe-mounted IMUs are not a valid method for detecting foot kinematic variables
Kleiner et al. (2018) [51]	Comparing TUG test total times measured by a wearable tri-axial IMU against an OMC system and a stopwatch	30 PD participants	BTS^®^ G-Sensor, BTS Bioengineering S.p.A., Milan, Italy. (1 IMU: low back)	BTS SMART System, BTS Bioengineering S.p.A., Milan, Italy. (8 cameras)	TUG time parameter	TUG test	ICC, ANOVA, and BA	The IMU showed excellent accuracy and precision in quantifying the TUG test completion times, similar to those obtained using the OMC system and a stopwatch
Al-Amri et al. (2018) [52]	Assessing the agreement between two systems for the measurement of the joint kinematics parameters	26 volunteer healthy subjects	Xsens MVN Biomech, Xsens Technologies BV, Enschede, The Netherlands. (7 IMUs: thighs, shanks, feet, and low back)	Vicon Motion Systems, Oxford Metrics Ltd., Oxford, UK. (10 cameras)	Joint kinematic measures of the hip, ankle, knee	Walking at normal speed on an 8 m straight pathway	CMC and R^2^	Despite not being interchangeable, joint angle parameters obtained from the two systems demonstrated excellent similarity in the sagittal plane and acceptable similarity in the frontal and transverse planes
Zago et al. (2018) [53]	Assessing the validity between IMU system and OMC system for the measurement of the ST gait parameters	22 PD participants	BTS^®^ G-Sensor, BTS Bioengineering S.p.A., Milan, Italy. (1 IMU: low back)	BTS SMART System, BTS Bioengineering S.p.A., Milan, Italy. (8 cameras)	Cadence, speed, stride length, stride time, step time, stance phase, swing phase, double support phase	Walking at self-selected speed on a 10 m walkway	Wilcoxon test, ES, RMSE, MAE, and PCC	The ST gait parameters detected by the IMU were mostly comparable to the output of the OMC system, except for speed
Teuf et al. (2018) [54]	Evaluating the agreement between two systems (IMU and OMC) for the measurement of the ST gait parameters	24 volunteer healthy subjects	Xsens MVN Biomech, Xsens Technologies BV, Enschede, The Netherlands (7 IMUs: shanks, feet, thighs, and low back)	OptiTrack—Motion Capture system, NaturalPoint Inc., Corvallis, OR, USA. (NS)	Step length, stride length, swing width, step width, step time, stride time, cadence, single support time, double support time, stance time, swing time, speed	Walking at normal speed on a 6 m straight pathway	RMSE, paired test, and BA	The IMU-based system demonstrated high validity for most parameters compared to the OMC system, except for step width and swing width
Teuf et al. (2018) [55]	Developing and validating an algorithm for joint kinematics measures estimation based on the IMU system compared with OMC system	28 volunteer healthy subjects	XSens MTw Awinda, Xsens Technologies BV, Enschede, The Netherlands. (7 IMUs: low back, shanks, thighs, and feet)	OptiTrack—Motion Capture system, NaturalPoint Inc., Corvallis, OR, USA. (13 cameras)	Joint kinematic measures of the hip, ankle, knee, pelvis	Walking for 6 min on a 10 m straight pathway at a self-selected speed	RMSE, ROME, CMC, and BA	The algorithm for calculating joint angles using the IMU system mounted on the lower limbs demonstrated strong/excellent agreement when compared to a standard OMC system
Fleron et al. (2019) [56]	Evaluating the accuracy of trunk speed extracted using an inertial motion system compared to an OMC system during steady walking	11 volunteer healthy subjects	Xsens MVN Biomech, Xsens Technologies BV, Enschede, The Netherlands. (17 IMUs: shoulders, arms, forearms, hands, thighs, shanks, feet, head, sternum, and low back)	Motion Capture system MA, Qualisys AB, Göteborg, Danmark. (8 cameras)	Trunk speed	Walking at self-selected speed on three pre-established pathways (1 × 1 m path, 2 × 2 m path and 2 × 3 m path)	RMSE, PCC, and ANOVA test	Close agreement between the IMU and the OMC system in detecting trunk speed was assessed during a standard walking task
Adamowicz et al. (2019) [57]	Evaluating the validity of novel sensor-to-sensor relative orientation and sensor-to-segment alignment algorithms by assessing performance in the estimation of hip joint angles in human subjects.	20 volunteer healthy subjects	Opal System, APDM Inc., Portland, OR, USA. (8 IMUs: feet, shanks, thighs, low back, and sternum)	Vicon Motion Systems, Oxford Metrics Ltd., Oxford, UK. (19 camera)	Joint kinematic measures of hip	Treadmill walking for 60 s at self-selected speeds	PB and RMSE	Close agreement was shown when comparing the MIMU-based method for estimating sensor-to-sensor relative orientation and hip joint angles with the OMC system
Amitrano et al. (2020) [58]	Assessing the validity of the ST gait parameters of novel wearable device SWEET Sock for remote health monitoring	3 volunteer healthy subjects	Prototype (2 IMUs: ankles)	BTS SMART System, BTS Bioengineering S.p.A., Milan, Italy. (6 cameras)	GCT, stance time, stance phase, swing time, swing phase, single support phase, double support phase, cadence, stride length, speed	Walking at normal speed on an 11 m straight pathway	Paired test, PCC, PB, and BA	The study revealed good agreement for temporal parameters such as gait cycle time and cadence, but not for spatial parameters, notably step length
Berner et al. (2020) [59]	Assessing the validity of an IMU system for measuring lower limb kinematic and ST gait parameters in people living with HIV (PLHIV) and HIV-seron negative participants (SNP)	8 PLHIVs and 8 SNPs	MyoMotion Noraxon system, Noraxon Inc., Scottsdale, AZ, USA. (7 IMUs: low back, feet, shanks, and thighs)	Vicon Motion Systems, Oxford Metrics Ltd., Oxford, UK. (8 cameras)	Step length, stride length, cadence, stance time, step time, single support time, double support time, stance phase, single support phase, double support phase, speed ROM of ankle, knee, hip, pelvis	Walking at self-selected speed along a 10 m pathway	RMSE, ICC, and BA	Good agreement was obtained between the IMU system and the OMC system for all kinematic and ST gait parameters, except for double support time and parameters expressed as a percentage of the gait cycle
Jordan et al. (2021) [60]	Assessing the validity of lower limb joint kinematic measures from the IMU system compared with OMC system during linear decelerations at various running speeds	1 volunteer healthy subject	Xsens IMU sensors, Xsens Technologies BV, Enschede, The Netherlands. (7 IMUS: feet, shanks, thighs, and low back)	Motion Capture system MA, Qualisys AB, Göteborg, Danmark. (11 cameras)	GCT, foot-CoM Joint kinematic measures of ankle, knee, and hip	ADA test	PCC, MD, ES, and TEE	High accuracy was obtained for Xsens IMU to detect ST parameters and hip and knee kinematics at low speeds, except for decelerations at higher speeds
Ziagkas et al. (2021) [61]	Evaluating the agreement between the PODOSmart insoles system and an OMC system.	11 volunteer healthy subjects	PODOSmart^®^ system, Digitsole SAS, Nancy, France. (inertial platform insole)	Vicon Motion Systems, Oxford Metrics Ltd., Oxford, UK. (10 cameras)	Stride length, stride time, stance phase, swing phase, circumduction, clearance, flat foot time, propulsion rate, propulsion time, cadence, speed, double support phase Joint kinematic measures of foot	Walking at normal speed on a 6 m straight pathway	ICC	Accurate measurements were obtained from the PODOSmart^®^ system compared to the Vicon system for temporal gait parameters but not for spatial parameters. Joint angle parameters showed poor or moderate accuracy
Saggio et al. (2021) [62]	Assessing the agreement of the ST features during walk using the IMU-based Movit System G1 compared with the camera-based Vicon system	8 volunteer healthy subjects	Movit System G1, Captiks Srl., Rome, Italy. (7 IMUs: low back, shanks, feet, and thighs)	Vicon Motion Systems, Oxford Metrics Ltd., Oxford, UK. (6 cameras)	Cadence, double support phase, single support phase, step length, step time, stride length, stride time, speed, stance phase, swing phase Joint kinematic measures of pelvis, hip, knee, ankle	Walking at self-selected speed on a 6 m walkway	RMSE, PCC, ε, ε%, and BA	The IMU system obtained accurate joint performance and excellent agreement in all ST parameters, except for knee varus/valgus and ankle inversion/eversion, step length, and double support
Simonetti et al. (2021) [63]	Developing and validating a wearable framework allowing the estimation of both the CoM acceleration and velocity from an optimal network of MIMUs	1 participant with transfemoral amputation	Xsens IMU sensors Xsens Technologies BV, Enschede, The Netherlands. (7 MIMUs: feet, shanks, thighs, and sternum)	Vicon Motion Systems, Oxford Metrics Ltd., Oxford, UK. (NS)	CoM acceleration, CoM velocity	Walking at self-selected speed along an 8 m pathway	RMSE and PCC	Strong agreement was obtained when comparing a network of five MIMUs with an OMC system in estimating CoM acceleration and velocity in a person with a transfemoral amputation
Romijnders et al. (2021) [64]	Assessing the shank-mounted IMU-based detection of GEs in different walking tasks and different mobility-limiting chronic diseases against an OMC system	11 older adults 14 PD participants 9 ST participants	MyoMotion Noraxon system, Noraxon Inc., Scottsdale, AZ, USA. (2 IMUs: shanks)	Motion Capture system MA, Qualisys AB, Göteborg, Danmark. (12 cameras)	Toe-off, heel strike	(1) Walking at self-selected speed along a 5 m pathway (2) Slalom trial (3) Stroop-and-walk trial	MAE, Wilcoxon test, recall, precision, F1	The shank-mounted IMUs showed good accuracy in detecting GEs during straight walking, except for curved walking tasks due to an increase in missed and false events
Piche et al. (2022) [65]	Assessing the validity of joint kinematic measures from IMU system with reference to the OMC system at different walking speeds	22 volunteer healthy subjects	iSen STT-IWS sensors, STT Systems Inc., San Sebastian, Spain. (11 IMUS: rearfoot, forefoot, shanks, thighs, low back, sternum, and trunk)	OptiTrack—Motion Capture system, NaturalPoint Inc., Corvallis, OR, USA. (9 cameras)	Joint kinematic measures of ankle, knee, and hip	Treadmill walking at three different speeds	RMSD, LCC, and BA	The comparison between the IMU iSen and the MOCAP OptiTrack showed good agreement at low speed and tolerable agreement at high speed
Rekant et al. (2022) [66]	Evaluating the validity of the joint kinematic measurement from the IMU-based Noraxon system compared with the OMC systems	10 volunteer healthy subjects	MyoMotion Noraxon system, Noraxon Inc., Scottsdale, AZ, USA. (7 IMUs: low back, thighs, shanks, and feet)	Vicon Motion Systems, Oxford Metrics Ltd., Oxford, UK. (14 cameras)	Joint kinematic measures of the hip, ankle, knee	Walking at self-selected speed across a tile floor	ICC and BA	No agreement was demonstrated, as kinematics in the sagittal plane performed better than in the frontal and transverse planes, while motion in the transverse plane at the ankle was unreliable
Bartoszek et al. (2022) [67]	Validating the joint kinematic measures during the Nordic walking gait recorded by an IMU-based system compared with an OMC system	1 volunteer healthy subject	MyoMotion Noraxon system, Noraxon Inc., Scottsdale, AZ, USA. (15 IMUs: trunk, arms, forearms, hands, neck, feet, shanks, thighs, and low back)	BTS SMART System, BTS Bioengineering S.p.A., Milan, Italy. (6 cameras)	Joint kinematic measures of the hip, ankle, knee, shoulder, elbow, wrist	Walking at velocity is preferred for the Nordic walking gait style for 12 m	PCC, BA, and SEE	The joint angle values obtained using MyoMotion were significantly higher or lower than the joint angle values obtained using BTS due to the presence of a constant systematic error
Choo et al. (2022) [68]	Evaluating the validity of the joint kinematic measurement from the Perception Neuron system with reference to a conventional OMC system	10 volunteer healthy subjects	Perception Neuron motion capture system, Noitom Ltd., Miami, FL, USA. (17 IMUs: NS)	Vicon Motion Systems, Oxford Metrics Ltd., Oxford, UK. (8 cameras)	Joint kinematic measures of the hip, ankle, knee	Walking at self-selected speed on a 3 m walkway	PCC, RMSE, and BA	The performances of PNS were good overall compared to the OMC, except only in hip flexion/extension during walking
Digo et al. (2022) [69]	Comparing three different IMU set-ups (trunk, shank, and ankle) to an OMC system for the evaluation of gait ST parameters in a healthy elderly population	16 volunteer healthy subjects	XSens MTx Awind, Xsens Technologies BV, Enschede, The Netherlands. (5 IMUs: trunk, shanks, and ankles)	OptiTrack—Motion Capture system, NaturalPoint Inc., Corvallis, OR, USA. (2 cameras)	Speed, stride time, step time, stance time, swing time	Walking at three different speeds on a 6 m straight pathway	PCC, RMSE, and BA	All the IMU configurations produced a good performance for GA; however, the trunk-IMU system seems to outperform the ankle-IMU and shank-IMU
Carcreff et al. (2022) [70]	Assessing the concurrent validity of a new IMU-based 3D lower-limb kinematics computation method on a healthy population against the OMC system	10 volunteer healthy subjects	Physilog 6S, GaitUp SA, Lausanne, Switzerland. (7 IMUs: low back, thighs, shanks, and feet)	Motion Capture system MA, Qualisys AB, Göteborg, Danmark. (20 cameras)	Joint kinematic measures of the hip, ankle, knee, pelvis, foot progression	Walking back and forth along the 10 m walkway at a spontaneous speed	RMSE, PCC, and ΔROM	The two systems are not completely interchangeable due to significant differences in joint kinematic measures along the frontal and transverse planes
Hellec et al. (2022) [71]	Evaluating the concurrent validity of step duration and step length recorded with an IMU embedded in smart glasses compared with an OMC system	20 volunteer healthy subjects	Prototype (1 IMU on eyeglasses)	OptiTrack—Motion Capture system, NaturalPoint Inc., Corvallis, OR, USA. (6 cameras)	Step time, step length	Treadmill walking at three different speeds	PCC and BA	Good agreement was assessed between the IMU embedded in the glasses and the OMC system to measure step duration and step length during gait assessment at different speeds
Romijnders et al. (2022) [72]	Assessing the validity of a deep learning approach for detecting GEs from an IMU placed on the lower leg in healthy YA, healthy OA, PD participants, MS participants, STR participants, cLBP participants, and others, compared to an OMC system	42 YA 22 OA 31 PD 21 MS 21 STR 9 cLBP 11 other participants	MyoMotion Noraxon system, Noraxon Inc., Scottsdale, AZ, USA. (4 IMUs: shanks and ankles)	Motion Capture system MA, Qualisys AB, Göteborg, Danmark. (12 cameras)	Toe-off, heel strike, stride time, stance time, swing time	Walking a distance of 5 m at three different self-selected speeds	BA and ε	Close agreement was assessed between the deep learning approach based on IMUs placed on the lower limbs and the OMC system for detecting ST parameters and GEs
Ricciardi et al. (2023) [73]	Evaluating the agreement between two systems for the measurement of the ST gait parameters in patients with PSP	15 PSP participants	Opal System, APDM Inc., Portland, OR, USA. (3 IMUs: low back and feet)	BTS SMART System, BTS Bioengineering S.p.A., Milan, Italy. (6 cameras)	Cadence, GCT, speed, stance phase, swing phase, stride length	Walking at normal speed on a 10 m straight pathway	Paired test, PB, and BA	The two systems are not completely interchangeable, due to two types of errors: a constant systematic error (cadence and GCT) and a proportional error (stance phase, swing phase, and stride length)
El Fezazi et al. (2023) [74]	Developing and validating a method for estimating knee kinematics during the TUG test using IMU devices compared to an OMC system	7 volunteer healthy subjects	XSens MTw Awinda, Xsens Technologies BV, Enschede, The Netherlands. (2 IMUs: shank and thigh)	Vicon Motion Systems, Oxford Metrics Ltd., Oxford, UK. (4 cameras)	Joint kinematic measures of knee	TUG test	Paired test, RMSE, PCC, and BA	No significant difference was shown in extracted kinematics parameters compared to the reference system, demonstrating strong agreement between the two methodologies
Brasiliano et al. (2023) [75]	Validating of three IMU-based algorithms (shank and foot set-up) for identifying GEs in children with ITW, both barefoot and while wearing a foot orthosis, compared with the OMC system	6 children with ITW	Opal System, APDM Inc., Portland, OR, USA. (4 IMUs: feet and shanks)	Vicon Motion Systems, Oxford Metrics Ltd., Oxford, UK. (7 cameras)	Toe-off, heel strike, stride length, swing time, stance time	Walking at self-selected speed	BA	The IMU-foot algorithm was the best for identifying heel strikes and estimating ST parameters, while the IMU-shank algorithm excelled at identifying toe-off
Pacher et al. (2023) [76]	Estimating the potential of multibody optimization to reduce errors in the lower-body kinematics obtained with IMUs compared with an OMC system	15 volunteer healthy subjects	Xsens IMU sensors Xsens Technologies BV, Enschede, The Netherlands. (7 IMUs: low back, thighs, shanks, and feet)	Vicon Motion Systems, Oxford Metrics Ltd., Oxford, UK. (18 cameras)	Joint kinematic measures of the hip, ankle, knee, and pelvis	Walking at self-selected speed along an 8 m pathway	RMSE, PCC, and ΔROM	Multibody optimization does not make a very significant contribution to improving lower-body kinematics obtained with IMUs

Abbreviation: ε = absolute error, ε% = absolute percentage error, ∆ROM = absolute difference in ranges of motion, ADA = acceleration–deceleration ability test, BA = Bland–Altman, CoM = center of mass, CMC = coefficient of multiple correlation, R2 = coefficient of determination, cLBP = chronic low back pain, foot-CoM = foot-center of mass, GEs = gait events, GCT = gait cycle time, ITW = idiopathic toe walking, ICC = intraclass correlation coefficient, IMU = inertial measurement unit, LR = likelihood ratio, LCC = Lin’s concordance correlation, MD = mean difference, MAE = mean absolute error, MS = multiple sclerosis, NS = not specified, ES = non-parametric effect size, OA = old adults, PCC = Pearson correlation coefficient, PD = Parkinson’s disease, PSP = progressive supranuclear palsy, PB = Passing–Bablok, RMSD = root mean square deviation, RMSE = root mean square error, ROME = range of motion error, STR = stroke, ρ = Spearman’s rank correlation coefficient, SEE = standardize typical error, TUG = timed up and go, TEE = typical error of estimate, YA = young adults.

**Table 2:** Distribution of subject impairments as reported in the studies included in this systematic review.

Impairment	References	Number of Subjects
Parkinson’s disease	[49]	11
	[51]	30
	[53]	22
	[64]	14
	[72]	31
Stroke	[64]	9
	[72]	21
Transfemoral amputation	[63]	1
Obese	[48]	8
HIV-positive	[59]	8
Multiple sclerosis	[72]	21
Chronic low back pain	[72]	9
Progressive supranuclear palsy	[73]	15
Idiopathic toe walking	[75]	6
Not specified condition	[72]	11

**Table 3:** Gait parameters computed in the studies included in this systematic review. The parameters are categorized based on their type, with the total number of parameters measured per category indicated in parentheses. The “Articles” column provides the reference for each measurement, while the “Total” column displays the overall count for each individual parameter.

Joint Kinematics Angles (117)					
Parameter	Total	Articles	Parameter	Total	Articles
ROM of foot	1	[50]	Shoulder abduction	1	[67]
ROM of ankle	3	[59,60,65]	Elbow flexion	1	[67]
ROM of hip	3	[59,60,65]	Wrist abduction	1	[67]
ROM of pelvis	1	[59]	Ankle min dorsiflexion	1	[65]
ROM of knee	3	[59,60,65]	Ankle min plantarflexion	1	[65]
Pelvic tilt	5	[45,55,62,70,76]	Ankle peak dorsiflexion	1	[60]
Pelvic obliquity	5	[45,55,62,70,76]	Ankle peak plantarflexion	1	[60]
Pelvic rotation	5	[45,55,62,70,76]	Hip peak flexion	1	[65]
Knee abduction	7	[52,55,62,66,67,70,76]	Hip minimum flexion	1	[65]
Knee rotation	7	[52,55,62,66,67,70,76]	Knee peak flexion	2	[60,65]
Knee flexion	9	[52,55,62,66,67,68,74,76]	Knee minimum flexion	2	[60,65]
Hip abduction	8	[52,55,57,62,66,67,76]	Heel strike angle	1	[61]
Hip rotation	8	[52,55,57,62,66,67,76]	Supination angle at heel-off	1	[61]
Hip flexion	10	[52,55,57,60,62,66,67,68,70,76]	Supination angle at heel strike	1	[61]
Ankle abduction	7	[52,55,62,66,67,70,76]	Supination angle at toe-off	1	[61]
Ankle rotation	7	[52,55,62,66,67,70,76]	Supination angle at toe-strike	1	[61]
Ankle flexion	8	[52,55,62,66,67,68,70,76]	Foot progression angle	1	[70]
Shoulder flexion	1	[67]			
**Spatiotemporal** (91)					
**Parameter**	**Total**	**Articles**	**Parameter**	**Total**	**Articles**
Swing time	5	[54,58,69,72,75]	Step width	1	[54]
Step time	7	[46,47,54,59,62,69,71]	Double support time	2	[54,59]
Step length	5	[47,54,59,62,71]	Single support time	2	[54,59]
Swing phase	5	[53,58,61,62,73]	Swing width	1	[54]
Stride length	9	[48,53,54,58,59,61,62,73,75]	Step count	1	[47]
Stride time	6	[48,53,61,62,69,72]	Trunk speed	1	[56]
Cadence	8	[48,53,54,58,59,61,62,73]	Circumduction	1	[61]
Speed	9	[48,53,54,58,59,61,62,69,73]	Flat foot time	1	[61]
Single support phase	3	[58,59,62]	Population rate	1	[61]
Stance phase	7	[48,53,58,59,61,62,73]	TUG time	1	[51]
Stance time	6	[54,58,59,69,72,75]			
GCT	3	[58,60,73]			
Double support phase	6	[48,53,58,59,61,62]			
**Center of Mass** (3)					
**Parameter**	**Total**	**Articles**			
Foot-CoM	1	[60]			
CoM velocity	1	[63]			
CoM acceleration	1	[63]			
**Gait Events** (9)					
**Parameter**	**Total**	**Articles**			
Heel strike	5	[47,49,64,72,75]			
Toe-off	4	[49,64,72,75]			

## Data Availability

Not applicable.

## Supplementary Materials

The following supporting information can be downloaded at: https://www.mdpi.com/article/10.3390/diagnostics15010036/s1, PRISMA 2020 Main Checklist. Reference [87] is cited in the Supplementary Materials.
